# DIGITAL STORYTELLING AMONG AFRICAN AMERICAN CAREGIVERS: EVALUATION OF THE INTERVENTION PARAMETERS

**DOI:** 10.1093/geroni/igae098.1105

**Published:** 2024-12-31

**Authors:** Abiola Keller, Aline Gubrium, Saharra Dixon, Bethani Bell, Dani Hart, Kristin Haglund

**Affiliations:** Marquette University, Milwaukee, Wisconsin, United States; University of Massachusetts Amherst, Amherst, Massachusetts, United States; University of Massachusetts Amherst, Amherst, Massachusetts, United States; Marquette University, Milwaukee, Wisconsin, United States; Marquette University, Milwaukee, Wisconsin, United States; Marquette University, Milwaukee, Wisconsin, United States

## Abstract

Digital storytelling (DST), a process involving creating 1–3-minute videos about an important moment in one’s life, is an innovative approach for data collection and intervention and it is imperative to understand storytellers’ perceptions of the process. This study explores the experiences of African American women caregivers enrolled in a DST intervention. The specific aims are to examine the acceptability, feasibility, and effectiveness of the DST process. Women who self-identified as African American, a caregiver (i.e., taking care of another adult), and agreed to attending all sessions were eligible to participate. Women were invited to participate in an interview via Microsoft Teams after each DST session. Interviews lasted approximately 15 minutes and were audio recorded and manually transcribed. Responses were examined for patterns and key content-related categories using inductive content analysis. In Cohort 1 (Spring 2023), five caregivers consented to participate in the DST sessions, three completed their story, and those three participated in interviews. In Cohort 2 (Fall 2023), five caregivers consented to participate in the DST sessions, four completed their story, and two participated in interviews. Women spoke about three distinct ways the DST process met their needs: 1) having a shared experience, 2) breaking the silence around caregiving, 3) learning and trying new things. Identified challenges included resistance to writing down their story and limited access to technology outside of the sessions. These findings suggest that DST may be used as an additional resource for supporting the well-being of African American women who care for older adults.

